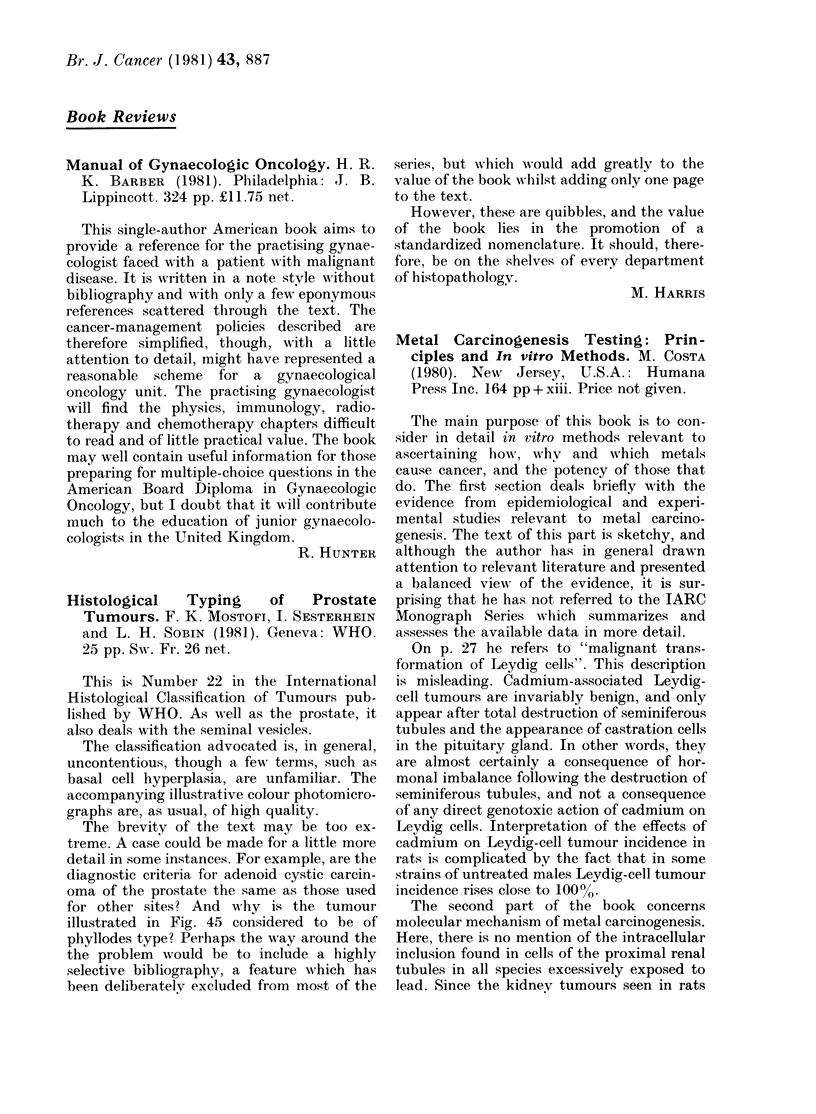# Histological Typing of Prostate Tumours

**Published:** 1981-06

**Authors:** M. Harris


**Histological Typing of Prostate Tumours.** F. K. MOSTOFI, I. SESTERHEIN and L. H. SOBIN (1981). Geneva: WHO. 25 pp. Sw. Fr. 26 net.

This is Number 22 in the International Histological Classification of Tumours published by WHO. As well as the prostate, it also deals with the seminal vesicles.

The classification advocated is, in general, uncontentious, though a few terms, such as basal cell hyperplasia, are unfamiliar. The accompanying illustrative colour photomicrographs are, as usual, of high quality.

The brevity of the text may be too extreme. A case could be made for a little more detail in some instances. For example, are the diagnostic criteria for adenoid cystic carcinoma of the prostate the same as those used for other sites? And why is the tumour illustrated in Fig. 45 considered to be of phyllodes type? Perhaps the way around the the problem would be to include a highly selective bibliography, a feature which has been deliberately excluded from most of the series, but which would add greatly to the value of the book whilst adding only one page to the text.

However, these are quibbles, and the value of the book lies in the promotion of a standardized nomenclature. It should, therefore, be on the shelves of every department of histopathology.

M. HARRIS